# Genetic and phylogenetic evolution of HIV-1 in a low subtype heterogeneity epidemic: the Italian example

**DOI:** 10.1186/1742-4690-4-34

**Published:** 2007-05-21

**Authors:** Luigi Buonaguro, Maria Tagliamonte, Maria Lina Tornesello, Franco M Buonaguro

**Affiliations:** 1 Lab of Viral Oncogenesis and Immunotherapy & AIDS Refer. Center, Ist. Naz. Tumori "Fond. G. Pascale", Naples, Italy

## Abstract

The Human Immunodeficiency Virus type 1 (HIV-1) is classified into genetic groups, subtypes and sub-subtypes which show a specific geographic distribution pattern. The HIV-1 epidemic in Italy, as in most of the Western Countries, has traditionally affected the Intra-venous drug user (IDU) and Homosexual (Homo) risk groups and has been sustained by the genetic B subtype. In the last years, however, the HIV-1 transmission rate among heterosexuals has dramatically increased, becoming the prevalent transmission route. In fact, while the traditional risk groups have high levels of knowledge and avoid high-risk practices, the heterosexuals do not sufficiently perceive the risk of HIV-1 infection. This misperception, linked to the growing number of immigrants from non-Western Countries, where non-B clades and circulating recombinant forms (CRFs) are prevalent, is progressively introducing HIV-1 variants of non-B subtype in the Italian epidemic. This is in agreement with reports from other Western European Countries.

In this context, the Italian HIV-1 epidemic is still characterized by low subtype heterogeneity and represents a paradigmatic example of the European situation. The continuous molecular evolution of the B subtype HIV-1 isolates, characteristic of a long-lasting epidemic, together with the introduction of new subtypes as well as recombinant forms may have significant implications for diagnostic, treatment, and vaccine development. The study and monitoring of the genetic evolution of the HIV-1 represent, therefore, an essential strategy for controlling the local as well as global HIV-1 epidemic and for developing efficient preventive and therapeutic strategies.

## Background

### HIV-1 genetic subtypes

**Figure 1:**
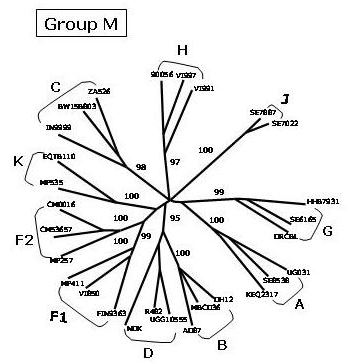
**Evolutionary relationships among non-recombinant HIV-1 strains**. The phylogenetic tree shows the subtypes of the M (main) HIV-1 group. The phylogenetic analysis has been performed on near-full length sequences and is based on neighbor joining method. The reliability of the internal branches defining a subtype has been estimated from 1'000 bootstrap replicates and the values are expressed as percentage.

The Human Immunodeficiency Virus type 1 (HIV-1) isolates are classified in three groups:**group M **(main), a **group O **(outlier) as well as a **group N **(non-M/non-O) [1-3]. The group M, responsible for the majority of infections in the HIV-1 worldwide epidemic, can be further subdivided into 10 recognized phylogenetic subtypes or clades (A – K, excluding E, which is actually a CRF), which are approximately equidistant from one another (Fig. 1).

HIV-1 phylogenetic classifications are currently based either on nucleotide sequences derived from multiple sub genomic regions (*gag, pol *and *env*) of the same isolates or on full-length genome sequence analysis. This approach has revealed virus isolates in which phylogenetic relations with different subtypes switch along their genomes. These *inter*-subtype recombinant forms are thought to have originated in individuals multiply infected with viruses of two or more subtypes. This results in the generation of several recombinants called "unique recombinant forms," or URFs [4]. When an identical recombinant virus is identified in at least three epidemiologically unlinked people, and is characterized by full-length genome sequencing, it can be designated as circulating recombinant forms (CRFs) [5-7]. The intra-genomic recombination appears to be a very frequent event and the CRFs account for 18% of incident infections in the global HIV-1 pandemic [8,9].

**Figure 2:**
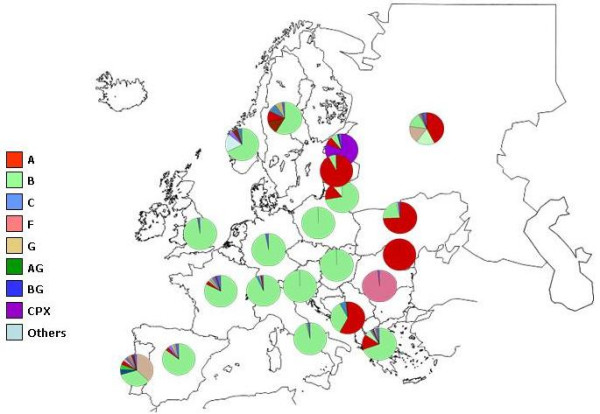
**Geographical distribution of HIV-1 genetic forms circulating in Europe**. Genetic forms predominant in the different European Countries are shown.

On a global scale, according to recent studies, the most prevalent HIV-1 genetic forms are subtypes A, B, C and CRF02_AG, with subtype C accounting for almost 50% of all HIV-1 infections worldwide. In Europe, in particular, subtype B is the circulating main genetic form, while subtype A viruses are predominant in east European countries formerly constituting the Soviet Union, where they are mainly transmitted among injecting drug users. Unlike all the surrounding Countries, Romania is characterized by an F subtype epidemic (Fig. 2).

### HIV-1 epidemic in Italy

**Figure 3:**
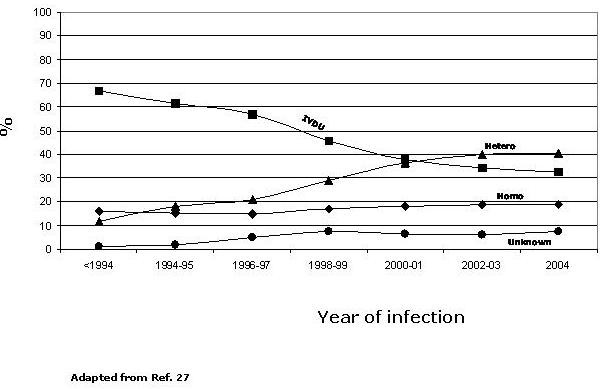
**Distribution of AIDS cases in adult population in Italy**. The percentage of AIDS cases for each risk group, over the HIV-1 epidemic, is indicated by lines. Unknown, indicates the undefined risk for infection.

Injecting drug users (IDUs) have been the most affected risk group during the first phase of the HIV epidemic in Italy and the HIV-1 B subtype, in accordance with other Western Countries, is the molecular form circulating among IDUs [10]. However, the annual percentages of AIDS cases reported in IDUs have gradually decreased to 32.3% in 2004 [11], in part as consequence of prevention programs [12,13]. In parallel, the AIDS cases reported in heterosexual individuals has continuously increased during the epidemic, becoming in 2004 the most prevalent risk factor for AIDS (40.4%) (Fig. 3) [10]. Similarly, in 2005 heterosexual contact accounts for over half (55%) of HIV infections newly diagnosed in the EU, nearly half (46%) of them were diagnosed in immigrants/migrants, primarily from sub-Saharan Africa, and most of these infections were acquired outside the EU (EuroHIV, 2006).

More than 10% of heterosexual individuals diagnosed with AIDS in Italy are either immigrants from endemic regions for HIV-1 (6.87%) or their Italian partners (3.03%). This epidemiological evidence, not considering all the HIV-1 infections derived also from traveling abroad, suggests that at least 10% of the viruses transmitted through heterosexual contacts could potentially belong to non-B subtypes and CRFs. This has been recently reported in other European Countries, with a higher prevalence due to an older tradition of immigration waves and much tighter historical as well as economic links with countries endemic for HIV-1 infection [14-22]

### Molecular evolution of the B-clade *env *sequences in the Italian epidemic

The biological relevance of genetic variations in the *env *gene is due to the central role of the envelope protein in the virus-host interaction. In particular, the V3 loop contains epitopes for strain-restricted neutralizing antibodies, it is a major determinant for viral tropism and co-receptor usage, and its orientation partially masks the CD4 and chemokine receptor binding sites [23-31].

The analysis performed including the B-subtype Italian sequences [32-45] has shown a progressive increase of nucleotide divergence in this region, increasing from 9.2% between isolates identified in the late 80's [46], to 17.51% between isolates identified in the early 2000's [33,45]. This closely resembles the expected evolution of a region under a strong immunological pressure during a long-lasting epidemic [45,47].

**Figure 4:**
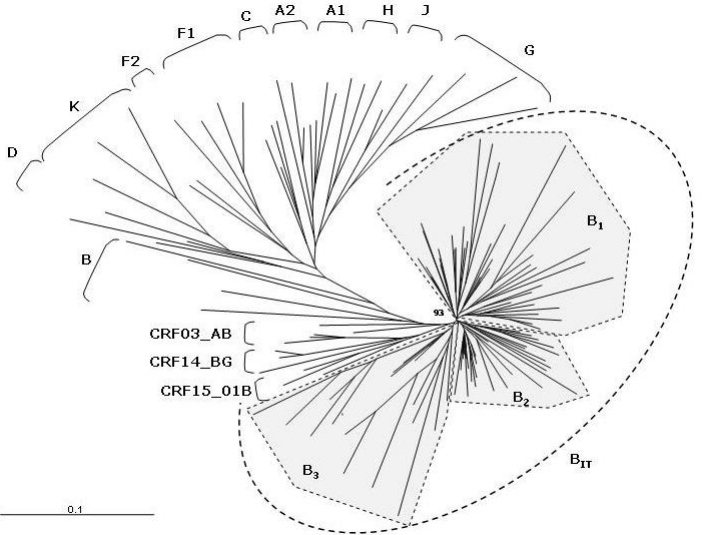
**Phylogenetic tree of HIV-1 *env *gene C2-V3 region from Italian B-clade isolates**. The C2-V3 *env *region (position 7001 to 7196 of HIV-1_HXB2_) of 143 Italian HIV-1 isolates, identified in the whole epidemic, has been aligned to reference sequences of all Group M subtypes, in order to generate the phylogenetic tree by the neighbor-joining method. The B_IT _indicates the "Italian branch" of the tree, which includes three major clusters B_1 _– B_3_. The reliability has been estimated from 1'000 bootstrap replicates. For editorial convenience, only the percentage value for the Italian Branch has been shown. All other values are > 90%.

Furthermore, a phylogenetic analysis performed on the same C2-V3 *env *region (position 7001 to 7196 of HIV-1_HXB2_) has shown the presence of an "Italian branch" where the HIV-1 isolates are distributed into three major clusters, each of them including several sub-clusters (Fig. 4). The 143 sequences derived from the different studies, selecting one sequence per patient deposited at the Los Alamos Database, do not form independent clusters and/or sub-clusters but are rather found inter-dispersed in the sub-clusters. This is likely due to the fact that the majority of the samples have been identified in Italy during overlapping periods in the early 90's. The distribution pattern of the sequences within the sub-clusters is not significantly associated to the risk factor for HIV-1 infection (IVDU, homo- or heterosexuality), by nonparametric Kruskal-Wallis test (p < 0,096). Moreover, the B_1 _cluster includes the majority of sequences identified in a broad time range, while the B_3 _cluster is prevalently based on recent sequences identified in our study. Moreover, as shown in Fig. 4, Italian B clade variants do not cluster with sequences from known "B clade-derived" CRFs.

### Rate of amino acid substitution and codon usage in the B-clade V3 *env *sequences

**Figure 5:**
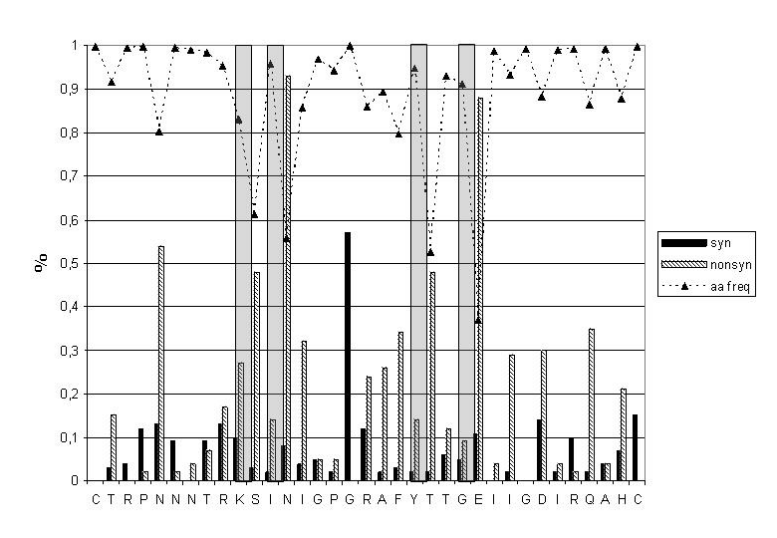
**Evolution pattern of the V3 loop**. The percentage of synonymous and non-synonymous substitutions in each of the 35 codons of the V3 Loop are indicated, together with the percentage of amino acid residue preservation at the specific position. The positions where the residue is found in < 80% of the sequences, are highlighted with light-gray boxes.

The B clade C2-V3 *env *sequences identified during the HIV-1 Italian epidemic have been subsequently analyzed for the frequency of synonymous and non-synonymous substitutions at each codon corresponding to the 35 aa forming the V3 loop of the *env *gene. The analysis has shown that very few codons (C_1_, R_2_, G_17_, G_28_, C_35_) are characterized by no substitutions or synonymous substitutions only, indicating the absolute conservation of those specific amino acid residues. In contrast, the vast majority of codons are characterized by a higher percentage of non-synonymous substitutions leading to amino acid changes. Nevertheless, the only residues found with a frequency < 80% at specific positions in the crown of the V3 loop are S_11_, N_13_, T_22 _and E_25_, although these do not seem to influence the binding of the gp120-CD4 complex to the CCR5 (Fig. 5). This is, in fact, mainly influenced by substitutions in the stem of the loop [48].

Furthermore, amino acid substitutions in the V3 loop show a significant uniform distribution in the HIV-1 sequences identified during the Italian epidemic, with the exception of the T-to-A_22 _substitution (within the tip of the loop) which is prevalent in the isolates identified in the early 2000's.

**Figure 6:**
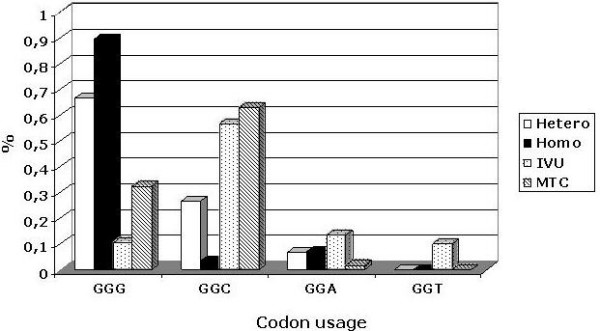
**Distribution of codons in risk groups**. The distribution of the four codons encoding the second glycine residue in the crown of the *env *gene V3 loop (GP**G**RAFYT) has been analyzed in HIV-1 sequences, identified in infected individuals with different risk practices.

The codon usage in the V3 region has been previously associated with HIV-1 isolates identified in patients with different risk factors. In particular, considering the second glycine at the tip of the V3 loop, the **GGG **codon has been associated with the homosexual risk group and the **GGC **codon with the IDU risk group [43,49-51]. In Italian B subtype sequences, the GGC codon is strongly associated with intra-venous transmission (p < 0.015), while the GGG codon is strongly associated with sexual (homo and hetero) transmission of HIV-1 (p < 0.007) (Fig. 6). The striking segregation of the GGC and GGG codons in the virus variants transmitted through different routes could be the consequence of different selections, including viral tropism, genetic bottlenecks or a founder effect.

### Non-B-clade *env *sequences in Italian epidemic

So far, during the entire HIV-1 epidemic in Italy, only seven non-B clade env sequences have been described, identified in heterosexual individuals (either immigrants from sub-Saharan Africa or their Italian partners) [44,45]; [33,34]. In particular, a very recent near-full length sequence analysis has shown that a HIV-1 isolate originally classified as A is actually close to the A3 sub-subtype and does not cluster in any of the known subtypes. It could potentially represents a novel sub-subtype, which needs to be confirmed with the identification of at least two additional related isolates in unlinked individuals [52].

### Molecular evolution of the B-clade *protease *sequences in Italian epidemic

The sequences relative to HIV-1 *pol *gene, and the *protease *region in particular, have been extensively analyzed and collected only from the year 2000, consequent to appearance of viral isolates resistant to protease inhibitors (PI), introduced as a component of anti-retroviral therapy (ART) combinations. This effect has made obvious the need to evaluate the resistant mutants to guide the choice of drug combinations in heavily drug-treated HIV-1-infected individuals as well as in recent treatment-naïve seropositive individuals.

The nucleotide divergence of the *protease *region during the HIV-1 epidemic in Italy has been evaluated including all the B-subtype Italian sequences from the published reports [53-64]. The analysis, unlike the analyses of the V3 *env *region, has shown a rather constant nucleotide divergence in this region (6.83% – 7.68%) over the 2000–2006 period. These results confirm that, also in a long-lasting epidemic, the *pol *genes (and the *protease *in particular) are not driven to genetic change by immunologic pressure.

**Figure 7:**
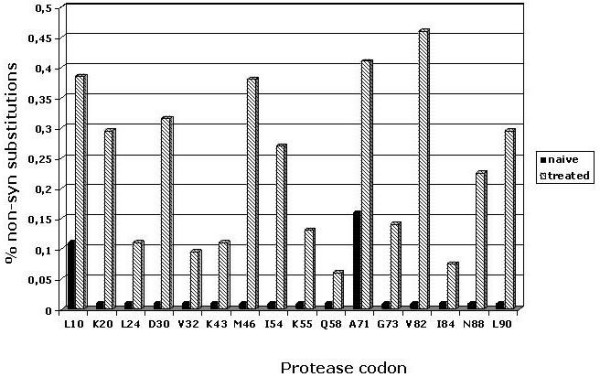
**Evolution pattern of the protease**. The percentage of non-synonymous substitutions in each of the protease codons were evaluated. The codons with the most significant difference between the sequences identified in naïve and ART-treated individuals are shown. The amino acid residues correspond to those found in the sequences identified in naïve individuals.

"Pharmacologic" pressure, instead, plays a significant role in the evolution of the *protease *gene by inducing the constant appearance and spread of mutant variants with degrees of drug resistance [65]. In this perspective, the synonymous and non-synonymous substitutions have been evaluated for the *protease *sequences described in Italy, showing the presence of "hot spot" in the 99 protease codons, where the frequency of non-synonymous substitutions has increased over the 2000–2006 period with the presence of PI drugs in the ART combination. In particular, sequences identified in ART-treated groups [54-56] showed a > 2.5 fold-increase in the frequency of non-synonymous substitutions at codons strongly associated with PI drug resistance, compared to sequences identified in a naïve group [62] (Fig. 7).

**Figure 8:**
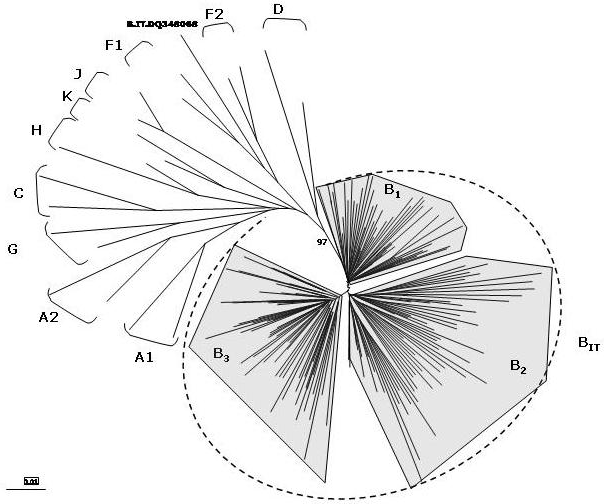
**Phylogenetic tree of HIV-1 *protease *gene from Italian B-clade isolates**. The *protease *region of Italian B-clade HIV-1 isolates, identified in the whole epidemic, has been aligned to reference sequences of all Group M subtypes, in order to generate the phylogenetic tree by the neighbor-joining method. The B_IT _indicates the "Italian branch" of the tree, which includes three major clusters B_1 _– B_3_. The reliability has been estimated from 1'000 bootstrap replicates. For editorial convenience, only the percentage value for the Italian Branch has been shown. All other values are > 90%.

The phylogenetic analysis performed on the *protease *region of the HIV-1 B-subtype Italian sequences showed, as for the *env *region, an "Italian branch" including three major clusters, each of them formed by several sub-clusters (Fig. 8). Also for the *protease *gene, as for the *env *C2-V3 region, sequences derived from the different studies do not form independent clusters and/or sub-clusters but are rather found inter-dispersed in the tree. Moreover, a distribution pattern based on the risk factor for HIV-1 infection (IVDU, homo- or heterosexuality) could not be assessed due to undisclosed demographic information. It is to be underscored that, as result of this phylogenetic analysis, the sequence 3193_1620A (Accession # DQ348068), deposited as B-subtype isolate [56], showed a strong phylogenetic link to the F1 subtype, suggesting that a revised classification of this isolate in the Los Alamos DataBase is appropriate.

The phylogenetic analyses, therefore, strongly suggest that, as for the *env *region, the protease region of the *pol *gene in HIV-1 B subtypes in Italy are derived from three main molecular ancestors, which have continuously evolved and spread among infected individuals during the epidemic.

### Non-B-clade *protease *sequences in Italian epidemic

**Figure 9:**
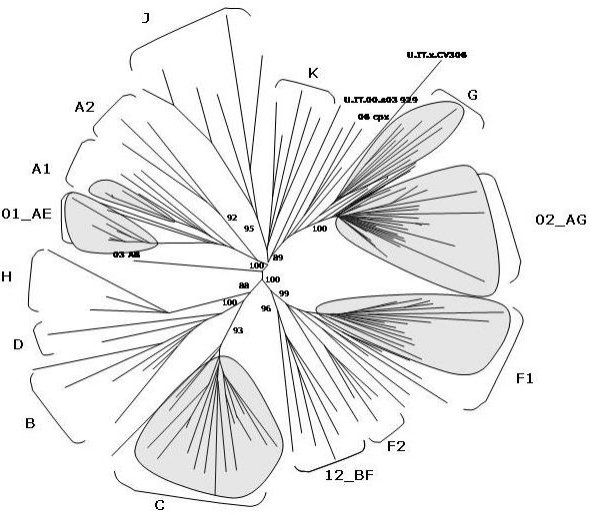
**Phylogenetic tree of HIV-1 *protease *gene from Italian non-B clade isolates**. The *protease *gene of non-B clade HIV-1 isolates, identified in the whole epidemic, has been aligned to reference sequences of all Group M subtypes, in order to generate the phylogenetic tree by the neighbor-joining method. The Italian sequences, in each subtype/CRF, are indicated by light-gray box. The reliability has been estimated from 1'000 bootstrap replicates and the values are expressed as percentage.

The non-B clade *protease *sequences, described in Italy over the 2000–2006 period [52,55,58,59,63,66,67], show *intra*-clade nucleotide divergences ranging from 3,34% (CRF01_AE) to 8,74% (F1), which are comparable to the divergence values observed for the B-clade sequences. Moreover, the phylogenetic analysis shows a limited evolution for each subtype, suggesting a recent introduction into Italy, although the limited number of isolates does not allow significant strong correlations to be made (Fig. 9).

### *Gag *sequences in Italian epidemic

**Figure 10:**
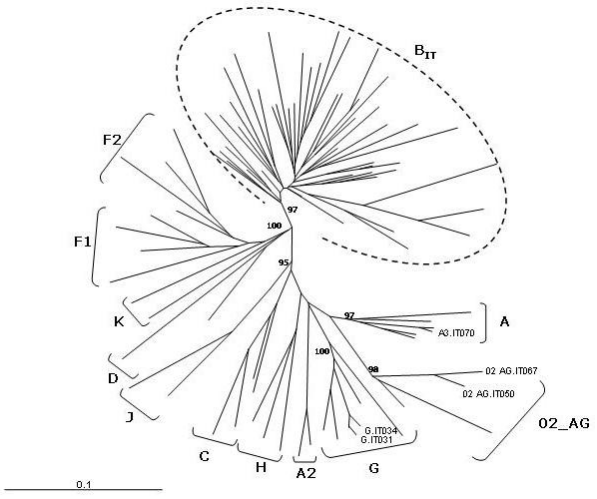
**Phylogenetic tree of HIV-1 gag p17 region from Italian isolates**. The gag p17 region of B as well as non-B clade HIV-1 isolates, identified in the whole epidemic, has been aligned to reference sequences of all Group M subtypes, in order to generate the phylogenetic tree by the neighbor-joining method. The B_IT_ indicates the "Italian branch" of the tree; the Italian non-B sequences are individually indicated. The reliability has been estimated from 1’000 bootstrap replicates and only values >90% are shown.

Nucleotide sequence analysis of the *gag *gene has not been a priority over the HIV-1 epidemic in Italy, and a very limited number of B as well as non-B clade sequences have been described [44,45,52,68]. A comprehensive phylogenetic analysis confirms the original subtype classification of the isolates and shows a distribution of the Italian B-subtype in different sub-clusters, where the sequences deriving from the different studies are found interspersed (Fig. 10). The lack of "cross-epidemic" sequences, however, does not allow inferences on phylogenetic evolution in *gag*.

## Concluding remarks

The B clade remains predominant and is circulating among all risk groups in the Italian epidemic, as observed all across Western European Countries [69]. Nevertheless, the structural genes of B subtype HIV-1 variants show a continuous spectrum of genetic diversification, although the currently circulating viruses appear to derive from a few early "founders". The introduction and the spread of non-B subtype HIV-1 isolates in the Italian epidemic, in contrast, appear to be still limited. In particular, as reported in other Western European countries, it is strongly associated with heterosexual transmission between local and immigrant/migrant partners. In this regard, it has to be mentioned that the general strategy of sequencing and performing phylogenetic analyses only on the *env *sub-genomic region, pursued in Italy and worldwide for many years, could have resulted in missing the identification of novel CRFs early in the Italian epidemic.

The Italian HIV-1 epidemic, therefore, represents a paradigmatic example of the European situation, being still characterized by low subtype heterogeneity. However, the slow introduction and diffusion of non-B subtypes in the population could progressively change the overall scenario and drive the need of adapting the diagnostic and treatment strategies currently used in European Countries.

## Authors' contributions

**LB **conceived of the study, analyzed data and drafted the manuscript; **MT **carried out the molecular genetic studies; **MLT **participated in the design of the study and performed the statistical analysis; **FMB **participated in its design, coordination and critically reviewed the manuscript. **All authors read and approved the final manuscript**
